# Review: innovation through research in the North American pork industry

**DOI:** 10.1017/S1751731119001915

**Published:** 2019-08-20

**Authors:** R. D. Boyd, C. E. Zier-Rush, A. J. Moeser, M. Culbertson, K. R. Stewart, D. S. Rosero, J. F. Patience

**Affiliations:** 1 Hanor Company, 128 W KY Ave, Franklin, KY 42134, USA; 2 Department of Animal Science, North Carolina State University, 120 W Broughton Dr, Raleigh, NC 27695, USA; 3 Rush Consulting, 373 Saint Martin Cir, Richmond Hill, GA 31324, USA; 4 Gastrointestinal Stress Biology Laboratory, Department of Large Animal Clinical Sciences, College of Veterinary Medicine, Michigan State University, 784 Wilson Rd, East Lansing, MI 48824, USA; 5 Global Product Development, Genus PIC USA, 100 Bluegrass Commons Blvd, Hendersonville, TN 37075, USA; 6 Department of Animal Sciences, Purdue University, 270 S Russell St, West Lafayette, IN 47907, USA; 7 The Hanor Company, 4005 E. Owen K. Garriott, Enid, OK 73701, USA; 8 Department of Animal Science, Iowa State University, 1221 Kildee Hall, Ames, IA 50011, USA

**Keywords:** genomics, seasonal infertility, amino acids, gut barrier function, functional ingredients

## Abstract

This article involved a broad search of applied sciences for milestone technologies we deem to be the most significant innovations applied by the North American pork industry, during the past 10 to 12 years. Several innovations shifted the trajectory of improvement or resolved significant production limitations. Each is being integrated into practice, with the exception being gene editing technology, which is undergoing the federal approval process. Advances in molecular genomics have been applied to gene editing for control of porcine reproductive and respiratory syndrome and to identify piglet genome contributions from each parent. Post-cervical artificial insemination technology is not novel, but this technology is now used extensively to accelerate the rate of genetic progress. A milestone was achieved with the discovery that dietary essential fatty acids, during lactation, were limiting reproduction. Their provision resulted in a dose-related response for pregnancy, pregnancy maintenance and litter size, especially in maturing sows and ultimately resolved seasonal infertility. The benefit of segregated early weaning (12 to 14 days of age) was realized for specific pathogen removal for genetic nucleus and multiplication. Application was premature for commercial practice, as piglet mortality and morbidity increased. Early weaning impairs intestinal barrier and mucosal innate immune development, which coincides with diminished resilience to pathogens and viability later in life. Two important milestones were achieved to improve precision nutrition for growing pigs. The first involved the updated publication of the National Research Council nutrient requirements for pigs, a collaboration between scientists from America and Canada. Precision nutrition advanced further when ingredient description, for metabolically available amino acids and net energy (by source plant), became a private sector nutrition product. The past decade also led to fortuitous discoveries of health-improving components in ingredients (xylanase, soybeans). Finally, two technologies converged to facilitate timely detection of multiple pathogens in a population: oral fluids sampling and polymerase chain reaction (**PCR**) for pathogen analysis. Most critical diseases in North America are now routinely monitored by oral fluid sampling and prepared for analysis using PCR methods.

## Implications

North American pork production has seen remarkable innovation in the past decade. Genetic improvement rate abruptly achieved a new trajectory, for pigs born late in 2013, because of genomic-enhanced selection. Insufficient intake of essential fatty acids by the mature sow limits litter size and is the primary cause of seasonal infertility. The list of health-promoting ingredients expanded to include xylanase, which improves viability. When oral fluid monitoring for population pathogens is combined with polymerase chain reaction technology, detection is rapid, affordable and the time frame to herd isolation is relatively short (days).

## Introduction

This article identifies the most significant research innovations that, in our opinion, have been applied by the North American pork industry in the past decade. They are primarily the result of leading edge research in North America. Research discoveries were not considered unless they were being integrated into practice, with the exception of gene editing for pathogen control, which is in the approval process.

Innovations that we identified as ‘milestone’ are diverse. Genome-enhanced breeding values resulted in an abrupt increase in the rate of genetic progress (e.g. piglet genome related to parent genes). Identification of a major gene for porcine reproductive and respiratory syndrome virus (**PRRSv**) and gene editing to ameliorate susceptibility to the virus is the most significant advance in PRRSv control in 25 years. Post-cervical artificial insemination (**PCAI**) is not new, but it has become a means to utilize elite sires to an even greater extent, in accelerating improvement (Safranski, 2008). Structure of the North American genetic industry facilitates the rapid dissemination of genetic improvement.

A remarkable advance in nutrition science resolved the long-standing problem of seasonal infertility for pigs. A direct relationship was observed between lactation fatty acid intake and reproduction (Rosero *et al*., 2016b), a finding that has been commercially validated in two southern regions of America. We also describe remarkable advances in our understanding of extended maternal influence on the development of neonatal innate immune capability and gut-barrier function, because this formed the scientific basis for a course correction from early weaning (12 to 16 days), for terminal pig production. This is a classic illustration where science was successfully applied to eliminate specific disease pathogens at the genetic nucleus and multiplication levels, but when it was applied to the commercial sector unexpected compromises emerged in piglet viability and growth.

Two important milestones were achieved to improve precision nutrition for growing pigs. The primary advance was the publication of the National Research Council (**NRC**) nutrient requirements for pigs (National Research Council, 2012), a collaboration between scientists from America and Canada. Precision nutrition advanced even further when ingredient description, for metabolically available amino acids and net energy (by source plant), became a private sector nutrition product. The past decade led to fortuitous discoveries of health-improving ingredients for growing pigs to market (xylanase, soybeans). Finally, a remarkable milestone was achieved with the convergence of technologies to detect the presence and to identify disease pathogens, easily and cost-effectively. These involved oral fluid sampling of populations and PCR technology, a means to rapidly identify animal disease to expedite herd isolation, especially African swine fever (**ASF**).

High-impact innovations likely to emerge in the North American pork industry during the next decade were also identified, and these are presented as a refereed supplement to this article (see Supplementary Material S1).

## Application of genome science

Genomic advances during the past two decades have been so significant that this field may be justly considered the science of the 21st century. Two important innovations were developed during the past decade that will profoundly impact North American pork production: (a) increasing genomic information to enhance selection accuracy and (b) gene editing for pathogen control. The former allows for more accurately defining parental genetic influence for each piglet, and this has abruptly advanced the rate of genetic improvement. The technology resulted in the first significant breakthrough in the control of PRRSv (Whitworth *et al*., 2016). This means to pathogen control resulted in the production of pigs that were shown to be completely resistant to PRRSv infection.

### Genomic-enhanced breeding values

The first widely utilized genomic test was applied in the 1990s to swine with the development of a genetic test to identify a HAL-1843 mutation of the ryanodine receptor gene (Fujii *et al*., 1991). This test identified animals that were likely to have an abnormal response to stress (two copies of the mutant allele) and revealed the potential of increased precision in livestock improvement that was possible with emerging technologies. However, this initial genomic tool depended primarily on finding closely linked associations between specific genetic marker and the phenotypic trait of interest (severe stress). The science of genomics continued to advance at a rapid pace through the late 1990s and early 2000s, while the underlying tools used for genomic testing began to dramatically evolve and sophisticate. A remarkable outcome of this evolution was development and commercialization of a new genotyping chip that allowed for rapid description of tens of thousands of genotypes on an individual test candidate (Ramos *et al*., 2009). This platform was the first application that allowed for a sufficiently expansive description of an animal’s genetic makeup using a robust chemistry platform (e.g. few genotyping errors, high call rates, automated platform). When this was combined with dramatically improved computational power, a new generation of technical opportunities emerged for genetic improvement.

**Figure 1:**
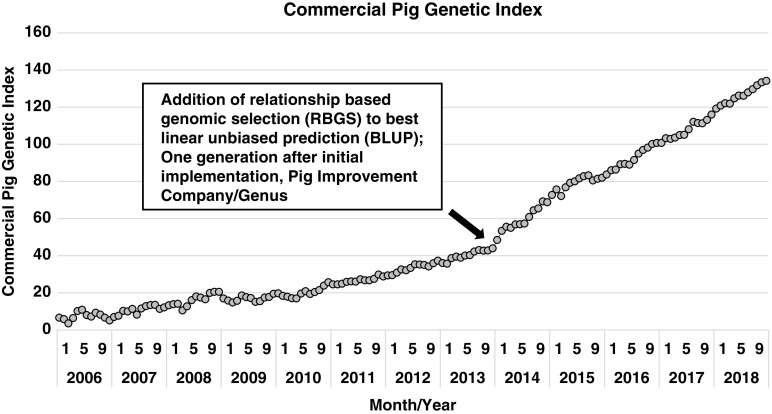
Genetic trend for index value of Pig Improvement Company (**PIC**) terminal sire × PIC F1 Camborough commercial pigs. Index value represents the overall genetic merit of an individual and is calculated as the weighted combination of economically important traits using its record and relatives. The steep increase in the genetic trend at the end of 2013 demonstrates the added value of using relationship based genomic selection (**RBGS**), rather than pedigree-only based relationships to calculate index values.

Expanding on the development of various statistical approaches to utilize this vast increase in information, Misztal *et al*. (2009) proposed an algorithm that utilized this expanding genomic information to more accurately describe the genomic segments that animals have in common (e.g. how piglet genome relates to each parent). This approach utilized best linear unbiased procedure (**BLUP**) models, but pedigree relationships were genomic-enhanced. From a practical perspective, this estimates the gene segments that any two animals have in common based on the genotype of each. This method is commonly referred to as single-step genomic evaluation or, in practice, relationship-based genomic selection (**RBGS**). Bundling of these technologies, the cumulative additive effects of enhanced computing power, expanded data capture (greater number of pigs captured in more diverse environments) and genomic technology, has abruptly increased the rate of improvement. This innovation bundle was implemented in the North American pork industry in early 2012 and simultaneously improved every trait and animal within the selection program. Delivering marginal improvement across the entire selection landscape enhanced annual progress by over 35% and over 50% for some traits that were either lowly heritable (e.g. pre- and post-wean viability) or sex-linked (e.g. total pigs born). The net result of this is shown in Figure 1, where a dramatic shift in commercial pig genetic index emerged one generation after the implementation of RBGS. In more accurately predicting the genetic index for each animal, the value declined for some individuals and increased for others. The net effect was to increase the range in selection index values.

State-of-the-art genome sequencing technologies have advanced to provide the next level in high-volume access to genomic data with remarkable changes in cost and efficiency. The first human genome draft was published in 2001, at a cost of approximately US$100 million. As with other technologies, evolving adjustments in the technical platform rapidly changed efficiency and cost, so that the cost to sequence the same genome is estimated to be approximately US$1,100 (Wetterstrand, 2019).

### Gene editing for pathogen control

The second innovation that emerged in North America is the development and exploration of gene editing as a tool to deliver precise genetic improvement. Gene editing is a method in which DNA is inserted, deleted, modified or replaced in the genome of a living organism. Unlike random insertions of early genetic engineering techniques, current genome editing technologies allow targeting to a specific location and to effect a precise change. Although multiple gene editors exist, identification and development of the clustered regularly interspaced short palindromic repeats (**CRISPR**)-Cas system appears to have enabled a quantum leap in efficiency and precision (Cong *et al*., 2013). The most significant swine application to date originated in North America with the announcement of pigs, developed at the University of Missouri via gene editing, that were resistant to the catastrophic disease PRRSv (Whitworth *et al*., 2016). In December 2015, the researchers announced the development of non-transgenic, gene-edited pigs that were resistant to PRRSv, which resulted from a small and precise edit to the *CD163* gene. The pigs, when subsequently challenged with the virus in a controlled challenge study, were no longer able to become infected, seroconvert and circulate the virus. They showed no viremia, antibody response or signs of clinical disease. These results have been replicated at multiple universities, across the globe, which reinforced the technical validity of the results (Burkard *et al*., 2017; Yang *et al*., 2018). There is beginning to be a steady stream of results generated in the area of precision gene-edited solutions to enhance the health, well-being and efficiency of the animal (Crispo *et al*., 2015; Carlson *et al*., 2016). An alternative to editing the major gene for PRRSv resistance is gene marker-based selection. Although this ‘natural’ approach is expected to be less effective, it does not require regulatory oversight.

### Natural selection for porcine reproductive and respiratory syndrome virus resistance

Genomic techniques to control PRRSv were applied in 2018 for gene editing and natural selection in North America. Gene edits to remove susceptibility to the PRRSv is the preferred method for disease resistance, but two barriers must be overcome: (a) US Food and Drug Administration clearance is required and demanding and (b) public acceptance is a concern. Genomic selection to identify pigs that have increased resistance is an alternative tactic because there is genetic variation in response to every disease in livestock, including PRRSv (Bishop, 2010). The difference between gene editing and natural selection for PRRSv is that gene edits are expected to result in complete resistance; natural selection for PRRSv would not result in complete resistance (Dunkelberger *et al*., 2017). The latter means that some pigs would still get sick, but they would have increased partial resistance to PRRSv: presumably carrying reduced viral load.

Since vaccinology and medical treatments have proven unsatisfactory in preventing PRRSv infection, a PRRSv host genetic consortium was formed to identify genetic associations to resistance (Lunney *et al*., 2011). They found a major gene, located on chromosome 4, that was associated with resistance to PRRSv. They also observed that, within this major gene, there is a specific genetic marker that could be used for selection. Incorporation of this Wageningen University Resistance gene (WUR) single nucleotide polymorphism genotype into an index scheme is believed to be a means of selecting for PRRSv resistance. If this proves to be true, this is a milestone for pig health because tools such as gilt acclimatization, vaccination and medication have proven ineffective in controlling PRRSv. Sow unit air filtration has evolved to become effective, but this method of reducing PRRSv infection is expensive and is not 100% effective.

## Reproductive technology–facilitated genetic improvement

An important milestone was achieved, during the past decade, to significantly increase the use of genetically elite sires. Post-cervical artificial insemination is used extensively in North America for that purpose. This technology is not novel, nor was it primarily advanced by North American scientists; however, recently it has been widely adopted to further accelerate the rate of genetic progress. Post-cervical artificial insemination technology was the outcome of strategic considerations to increase elite sire use. The first is to reduce the number of inseminations required to establish pregnancy. The second is to reduce the number of sperm cells used for each insemination, so that a boar’s ejaculate can be used to inseminate more females. The first objective is deliverable using a technology called OvuGel®. This technology is a method of delivering an ovulation synchronizing molecule (vaginal not intramuscular injection). It is commercially available in North America and delivers on single-fixed timed artificial insemination (**SFTAI**) to achieve pregnancy from one insemination. This technology was developed as a novel triptorelin (GnRH agonist) gel formulation, delivered intravaginally to weaned sows to induce ovulation (reviewed by Knox *et al*., 2018).

### Post-cervical artificial insemination

In order to reduce the number of sperm cells per insemination dose, sperm needs to be deposited closer to the site of fertilization in the female’s reproductive tract. Intrauterine artificial insemination, better known as PCAI, is a process by which semen is deposited beyond the cervix and into the uterine body. Conventional artificial insemination (**AI**) deposits semen in the cervix and requires 3 to 5 billion sperm cells per insemination dose to achieve acceptable fertility. The reason for the large quantity of sperm cells is because the cervix filters sperm, thereby reducing the number of cells that make it to the site of fertilization. Theoretically, PCAI, coupled with a reduction in total sperm dose, is expected to increase the number of piglets born from each boar by 16 000 per year when compared to conventional AI. The advantage of PCAI is estimated to be 23 000 compared to pen mating (Safranski, 2008). This technology is very important to genetic nucleus farms, but a 2007 survey of US sow farms reported that 6% were using PCAI (Knox *et al*., 2013). This increased to more than 40% in 2017 (Stewart K.R., unpublished research). Growth of PCAI in Canada appears to be similar or slightly less, based on PCAI catheter sales.

The adoption of PCAI was initially driven by the technology’s ability to facilitate an increase in the number of offspring produced from superior sires. However, as farms began to adopt PCAI, other economic benefits were identified. The process of performing conventional AI is labor intensive; multiple technicians are required, and each insemination takes 3 to 10 min. Post-cervical artificial insemination reduced the amount of time to inseminate each sow to about 1 min or less which reduced the amount of labor required. Large systems utilized this benefit to reduce the number of breeding technicians and to redirect that resource to the farrowing room. Although PCAI insemination has been increasing, it has not been equally accompanied by reductions in sperm content in the insemination dose. Of the 40% of farms that identified as utilizing PCAI in 2017, only 16% reported using low-dose semen technology (Stewart K.R., unpublished research). Adoption of a new technology requires that it perform equal to or better than the current method and PCAI needed to be proven in practice. Research attempting to determine a recommended sperm number for insemination produced equivocal results (Watson and Behan, 2002; Rozeboom *et a*l., 2004; Mezalira *et al*., 2005; Hernandez-Caravaca *et al*., 2012). It appears that PCAI dose concentrations of approximately 1 billion and below tend to decrease fertility.

### Deep uterine insemination

In order to further reduce sperm dose to less than 1 billion, deep uterine insemination (**DUI**) may be required; semen is deposited at the anterior end of the uterine horn and closer to the fertilization site in the oviduct. Prior to this, we need to understand the possible economic benefit of further leveraging superior sires. Fast Genetics (Deforest, WI, USA) estimated that DUI, performed with 500 million cells per dose, could produce 21 000 additional pigs per boar per year compared to PCAI with 1 billion cells per dose (Willenburg and BeVier, 2017). A recent commercial study showed that when SFTAI was combined with DUI, to deliver sperm concentrations ranging from 600 to 75 million (600, 300, 150, 75), pregnancy rates were similar but litter size decreased as sperm number declined below 600 million (Knox *et al*., 2019).

## Reproductive nutrition – essential fatty acids and seasonal infertility

A milestone was achieved with the discovery that dietary essential fatty acids (**EFA**), during lactation, are important to subsequent reproduction, exhibiting a dose-related response for achieving and maintaining pregnancy, as well as litter size. The dietary requirement is progressively more important with advancing reproductive cycles. This may be due to a steady depletion of EFA with each reproductive cycle (Rosero *et al*., 2016a and 2016b). A frank deficiency of parent EFA (linoleic acid, α-linolenic) is also more apt to occur during heat stress, when feed intake is reduced, and by feeding diets with no EFA sources added. This nutritional finding has proven successful against the problem of seasonal infertility in pigs in North America. Consequently, this technology is gaining adoption in North America.

### Seasonal infertility

Concurrent with the progressive increase in pig output and potential for nutrient deficit is the matter of seasonal infertility. This phenomenon is expressed in various ways, but chief among them is failure to maintain pregnancy. Upon weaning, sows may exhibit slower return to estrus and conceive, but pregnancy is interrupted (Peltoniemi and Virolainen, 2006). In practice, litter size also tends to decline. In North America, seasonal infertility is associated with mating in mid to late summer. While it has been associated with environmental factors, such as photoperiod, compromised reproduction also coincides with extreme heat stress (Ross *et al*., 2017). An initial response to heat stress is the reduction in feed intake. This intake suppression is believed to be a means to reduced fertility. We hypothesized that body EFA reserves become depleted by the reduction in intake combined with marginally deficient lactation diets over successive reproduction cycles, with this being exacerbated by gestation diets that do not recover the prior lactation EFA deficit.

### Essential fatty acid intake in lactation and reproduction

The role of EFA (linoleic acid, C18:2n-6; α-linolenic acid, C18:3n-3) in reproduction includes alteration of ovarian follicle and embryonic development, of hormone precursors important to reproduction, and pregnancy recognition and maintenance via cell signals (Thatcher *et al*., 2010). We determined with lactating sows that were nursing 12 pigs during summer heat stress that a practical lactation diet with no added lipid has a profound negative balance of linoleic acid and an apparent deficit in α-linolenic acid. This coincided with reduced sow fertility (farrowing rate < 75%, culling rates > 25% of weaned sows). This reduction in fertility seemed to be increasingly important with advancing sow age, presumably because of a progressive reduction in the body EFA pool over successive lactations (Rosero *et al*., 2016a). We computed the balance of EFA for six published studies, where diet EFA intake and milk EFA output was available (Rosero *et al*., 2015a), and arrived at a comparable imbalance between intake and output (linoleic acid, −25.5 g/day; α-linolenic acid, −2.8 g/day).

Moreover, we conducted a dose-response assay to determine the levels of both EFA (linoleic acid, C18:2n-6; and α-linolenic acid, C18:3n-3) required by the lactating sow for maximum subsequent reproduction (Rosero *et al*., 2016a). Both EFA were studied simultaneously because they have opposing functions, and increasing one EFA alters metabolism and physiological function of the other (Sprecher, 2000). Each EFA benefits reproduction through different modes of action. Provision of 0.45% dietary α-linolenic acid was the most effective dose (0.15%, 0.30%, 0.45%) in eliciting a rapid return to estrus and achieving the highest retention of pregnancy. However, it did not improve litter size. Supplemental linoleic acid improved total pigs born in a linear manner (13.2, 13.8 and 14.0 total pigs born/litter for 2.1%, 2.7% and 3.3% linoleic acid, respectively). Although supplemental linoleic acid improved litter size of first litter sows, the beneficial effects were more evident for older sows (litters 3 to 5).

Based on these findings, and especially for mature sows, a minimum dietary intake of both parent EFA was required to achieve rapid return to estrus, ability to conceive and maintain pregnancy and improved litter size. We concluded that a minimum dietary intake of 10 g of α-linolenic acid/day, simultaneous with a minimum of 125 g of linoleic acid/day should be provided to >98% of the sow population with the level being related to population daily intake. In this study, this equated to approximately 0.45% and 3.1% α-linolenic and linoleic acids, respectively (Rosero *et al*., 2016a). Since linoleic acid intake during lactation exhibited a dose-response increase in litter size and for pregnancy maintenance, we studied this in greater detail by using data from three studies, involving 543 mature sows (parities 3 to 5; Rosero *et al*., 2016b).

### Estimating essential fatty acid requirement for lactating sows

**Figure 2:**
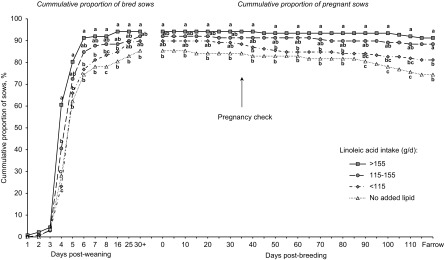
Effect of dietary linoleic acid intake during lactation on subsequent reproduction of sows (*n* = 84 sows fed diets with no added lipids; *n* = 152, 163 and 144 sows for <115, 115 to 155, and >155 g/day of linoleic acid intake, respectively) represents the cumulative proportion bred and pregnant sows relative to the number of sows weaned (SEM = 2.9). Analysis included a total of 543 mature sows (litters 3 to 5) from three studies. Sows fed diets without added lipids consumed 84.4 + 20.3 g/day of linoleic acid. Means represented by symbols without a common letter are different (*P* < 0.05). Figure reproduced from *Journal of Animal Science and Biotechnology*, BioMed Central Publishing, Creative Commons Attribution 4.0 International License (Rosero *et al*., 2016b).

Portraying the reproductive data as a continuum from weaning to farrowing was clarifying, in that patterns emerged to improve our understanding of increasing linoleic acid dose (Figure 2). Curves show the cumulative percentage of sows bred and maintaining pregnancy in relation to total sows weaned. Sows fed the reference diet (no added lipid) were more delayed in expressing estrus, and fewer did so. The proportion of weaned sows that were mated and confirmed pregnant was 84.4%, but only 74.4% of weaned sows farrowed. On the other hand, sows that consumed more than 115 g/day of linoleic acid exhibited an improved pattern, with approximately 90% bred (by day 8 post-wean) and maintained as pregnant through to farrowing. Thus, dose-related increases in linoleic acid intake produced dose-responsive improvements in how rapidly and the extent to which mature sows returned to estrus, pregnancy level and the ability to maintain pregnancy. Although consuming more than 155 g/day in lactation did not statistically improve reproduction (*v*. 115 g/day), neither was it deleterious. This pattern of improved reproduction coincided with incrementally reduced rates of culling for reproduction dysfunction (Rosero *et al*., 2016a).

**Figure 3:**
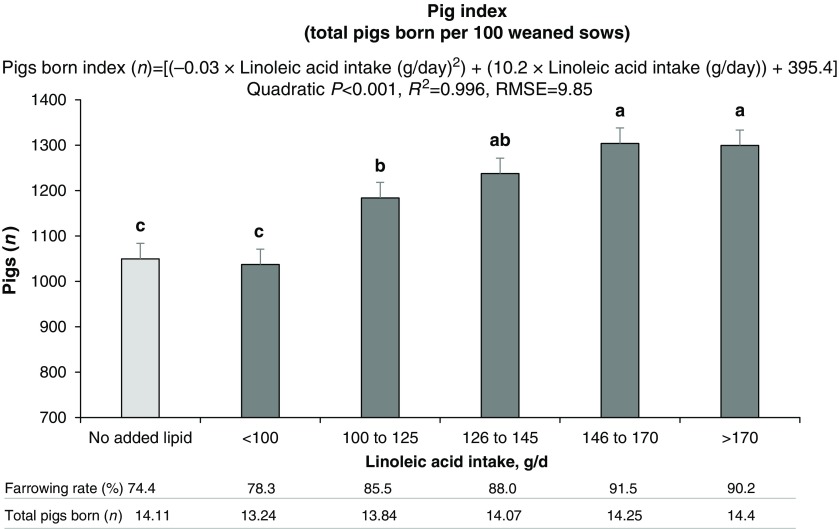
Effect of linoleic acid intake during lactation on pigs born index. This variable represents the total number of fully formed pigs born per 100 weaned sows and was calculated by multiplying subsequent farrowing rate (sows farrowed: weaned) by total number of pigs born/litter. Sows fed diets without added lipids served as control (84.4 + 20.3 g/day of linoleic acid). Means represented by bars without a common letter are different (*P* < 0.05). Figure reproduced from *Journal of Animal Science and Biotechnology*, BioMed Central Publishing, Creative Commons Attribution 4.0 International License (Rosero *et al*., 2016b).

We employed a measure (pig index) to refine our estimate of the linoleic acid dose response maximum (Figure 3). Pig index is the multiple of farrowing rate and total fully formed pigs born, and it quantifies the total pigs born per 100 sows weaned. The marginal difference when moving from 100 to 125 g/day to 126 to 145 g/day is 54 pigs/100 sows weaned. Moving from the latter to 146 to 170 g/day further improved marginal pigs born by 68. The regression equation in Figure 3 is the basis for computing optimum profit linoleic acid intake, but feeding to deliver less than 125 g/day, for perhaps >98% of the population, is a suggested minimum. This dose-response assay is the first estimate, to our knowledge, of the linoleic acid requirement for reproduction in any species. This estimate will vary with (a) age of sow, being greater for older sows compared to younger sows, and (b) EFA pool recovery in pregnancy. Proper implementation of EFA estimates requires knowledge of seasonal lactation intake and the variance around intake. These findings demonstrate for the first time that summer infertility can be largely mitigated by an important nutritional modification under moderate heat stress.

### Extreme and prolonged heat stress and seasonal fertility

We anticipate that seasonal infertility may still manifest in geographical regions with more ‘severe’ heat stress than encountered during our studies (ca. 36° latitude). Endocrine changes suppress oocyte development and survival, as well as embryo viability (Hansen, 2009); likewise, other physiological processes such as intestinal barrier dysfunction may occur due to localized hypoxia (Ross *et al*., 2017). Nevertheless, providing the required daily intake (g/day) for each EFA is expected to moderate against the most extreme reduction in reproduction.

## Immune development and gut barrier function in weaned pigs

### Wean age and gut development

The weaning period has long been considered the most stressful phase of swine production. In contrast to nature where weaning is a gradual process nearing completion between 3 and 4 months of age, weaning in swine production is abrupt and occurs between 2 and 4 weeks of age when many physiological systems, such as the gastrointestinal and immune systems of the piglet, are relatively immature. As a result, the post-weaning period has been associated with reduced feed intake and performance concurrent with increased disease susceptibility.

### Segregated early weaning: upside and downside

The original work by Alexander *et al*. (1980) proved that very early medicated weaning at 5 to 10 days of age and complete separation from the sow population could reduce the vertical transmission of certain pathogens from the sow to offspring, and thus serve as an elimination strategy for pathogens such as *Actinobacillus pleuropnemoniae*, *Haemophilus parasuis* and transmissible gastroenteritis virus (**TGEv**). This led to the movement toward segregated early weaning (**SEW**) and medicated early weaning (**MEW**) programs in the 1980s into the 1990s. Weaning age in these programs ranged between 10 and 21 days of age (Harris, 1990; Connor, 1992; Wiseman *et al*., 1992; Fangman and Tubbs, 1997). This pathway to disease elimination resulted in multiple site production, which was formalized as Isowean technology (Harris, 2000): two production sites for complete separation of weaned pigs from the sow herd as a constant, but three site systems were used by some to further separate nursery and finish phases. Unfortunately, this method of disease ‘elimination’ and minimization was not adequately studied for possible long-term downside on growth and viability.

### Unintended consequences of very early weaning (12 to 16 days)

While the immediate benefits of early weaning for specific pathogen elimination were realized, especially for genetic nucleus programs, commercial production suffered with decreased growth and viability (Patience *et al*., 2000). Commercial pigs easily developed diarrhea, needed increased levels of medication and experienced higher mortality and morbidity. This was confirmed by Main *et al*. (2004), who showed that increasing wean age (from 12 to 21.5 days of age) resulted in linear improvements in wean-to-finish performance and viability. The article by Cabrera *et al*. (2010) also demonstrated that the sow influences the performance of her progeny beyond the colostrum period, and in a profound way. Pigs reared by their mother for 20 days grew more efficiently, had greater loin muscle depth and tended to have fewer pigs die and removed for medical treatment than their 14-day counterparts, even though high health conditions existed.

**Figure 4:**
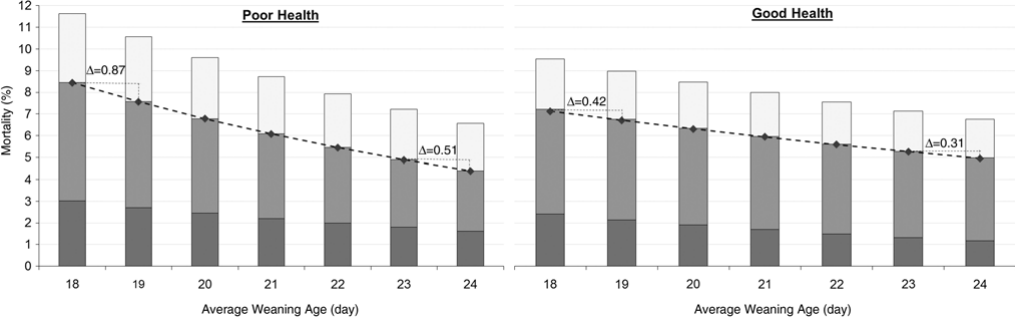
Impact of increasing weaning age from 18 to 24 days on percentage of pigs sold to off-grade market (or cull) and mortality of pigs weaned under (a) poor and (b) good health conditions. Bars and symbols represent estimated means obtained by using nonlinear (Poisson) regressions for mortality and culls. The darkest grey bars represent mortality from weaning to 7 weeks post-weaning (nursery period). The light gray bars represent mortality from 8 weeks post-weaning to marketing (finish period), and the line defines wean-to-finish mortality. The clear bars, above the mortality line, represent cull pigs below minimum full-value weight. The regressions estimate greater impact of increasing weaning age on pigs weaned at younger ages and during poor health conditions. The nonlinear regression for wean-to-finish mortality in poor health is Mortality (%) = Exponential [4.10 + (−0.109 × wean age)], *P* = 0.003, while the regression for good health is Mortality (%) = Exponential [3.05 + (−0.0601 x wean age)], *P* = 0.04 (adapted from Rosero *et al*. 2016c).

Field validation of these concepts was conducted by Rosero *et al*. (2016c), where the dynamic of changing health over time and with multiple sets of pigs was used. They studied the impact of wean age in a large population of animals (143 weaned groups; 1139 to 2725 pigs/group) over a 4-year period and showed that incremental increases in wean age (18 to 24 days) improved wean-to-finish performance and reduced mortality. Moreover, this study revealed that the negative impact of early wean age depended on health status of their mothers. This became clear with disease infection (PRRSv, porcine epidemic diarrhea virus (**PEDv**); Figure 4). This is also supported by McLamb *et al*. (2013), where early weaned pigs (16 to 17 days wean age) exhibited more severe growth reductions, diarrhea and intestinal injury when faced with subsequent infectious challenge with F18 *Escherichia coli* later in the nursery period, compared with pigs weaned at 22 days of age. Given the experience of the commercial sector, the industry has moved back to an older wean age, especially to eliminate weaning of extremely young litters (National Pork Board, 2013 and 2018).

### Understanding the biological mechanisms of lifetime performance and disease risk associated with early weaning

**Figure 5:**
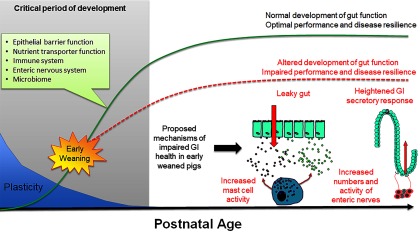
Impact of early weaning on long-term gut development in the pig. The early postnatal period is characterized by extensive development of critical system and gut functions (inset) and high plasticity. Development of gut function during this period shapes long-term gastrointestinal (**GI**) development, function and health (green line). Early weaning is a significant stressor causing intestinal injury (e.g. increased intestinal permeability, inflammation) that alters normal gut development leading to impaired performance and decreased disease resilience (red line). Proposed mechanisms underlying early weaning–induced intestinal dysfunction include increased mast cell activation, intestinal permeability (‘leaky gut’), heightened enteric nervous system activity and gut secretory pathways.

During the first 3 to 4 months of postnatal life, the gastrointestinal (**GI**) system in the pig undergoes dramatic development including the establishment of the epithelial barrier and digestive functions, microbiome colonization and development of the enteric nervous and immune systems (Pohl *et al*., 2015). Importantly, the GI system exhibits a high degree of plasticity during this period, and thus environmental factors (e.g. stress, intestinal injury, pathogen challenge) can disrupt the normal development of GI functions leading to long-term changes in gut health and disease resilience (Figure 5). There have been a large number of studies investigating the short-term impacts of weaning on intestinal injury, which is characterized by increased intestinal permeability and a spike in inflammation occurring within the first days of weaning (Bailey *et al*., 2005; Lalles *et al*., 2007; Moeser *et al*., 2007a). Thus, intestinal injury occurs in a peak period of GI development and programming, but the long-term effect on GI function has only recently been fully studied. Investigations using split-weaned litters allowed a comparison of gut developmental trajectories between early weaned (weaned at 18 days) and their later weaned littermates (weaned at 28 to 30 days). This revealed key insights into the long-term impact of early weaning on gut development, potential disease risk and performance reductions. Early weaning alters the normal development and function of the GI epithelial barrier, enteric nervous system and mucosal innate immune responses (Medland *et al*., 2016; Moeser *et al*., 2017; Pohl *et al*., 2017), which coincides with diminished performance and resiliency to later-life stress or infectious challenges. Disturbance in intestinal barrier function, measured as increased intestinal permeability or ‘leaky gut’, is significant as increased GI permeability allows an excessive leakage of antigens across the intestinal epithelium, which incites chronic mucosal inflammation and impaired gut function. Loss of barrier function is thought to be a major predisposing factor in a number of human stress-related diseases, including irritable bowel syndrome (**IBS**) and inflammatory bowel disease. Medland *et al*. (2016) showed that early weaned pigs have more numbers of enteric nerves and heightened nerve-mediated secretory activity compared with later weaned pigs. Heightened intestinal permeability and GI secretory function coincided with intermittent, chronic diarrhea in early weaned pigs (Porras *et al*., 2006; Barbara *et al*., 2011; Pohl *et al*. 2017). Early weaned pigs exhibited a suppressed mucosal innate immune response and more severe clinical disease and intestinal barrier injury in response to a nursery challenge with F18 *E. coli* (McLamb *et al*., 2013; Li *et al*., 2019).

Studies investigating the mechanisms driving these long-term alterations in GI showed that the GI mucosa from early weaned pigs often has a similar morphological appearance (e.g. villus height, inflammatory lesions, crypt depth) compared with later weaned pigs. However, a distinct histological feature reported in early weaned pigs was an increase in the number of intestinal mast cells and level of mast cell mediators such as tryptase (Smith *et al*., 2010; Pohl *et al*., 2017). Moreover, administration of the mast cell stabilizing drug, sodium cromolyn, either prior to weaning or in grower pigs, reduced intestinal permeability in early weaned pigs (Moeser *et al*., 2007b; Smith *et al*., 2010; Mereu *et al*., 2015). Together, these studies demonstrate that increased mast cell activity is critical to early and long-term intestinal permeability disturbances in the early weaned pig. The specific GI alterations observed in early weaned pigs are remarkably similar to those of human GI functional disorders such as IBS in which stress, intestinal permeability and heightened mast cell activity are known to be key mechanisms in symptom onset.

Early wean age disturbances may go beyond the GI tract. Furthermore, it is conceivable that early weaning could also impact organ systems other than the GI tract, such as the respiratory system. As discussed earlier, Rosero *et al*. (2016c) showed that performance and mortality of early weaned pigs were more negatively impacted during subsequent natural exposure to PRRSv, a predominantly respiratory pathogen. Epidemiological studies in humans and early life stress models in rodent (e.g. neonatal maternal separation stress) show a link between early life stress/adversity and long-term development of lung immunology, function and respiratory disease risk later in life (Bhan *et al*., 2014; Hupa *et al*., 2014; Lee *et al*., 2017).

## Innovations in precision nutrition

Two important milestones were achieved to improve precision in matching diet input to production needs for growing pigs during the past decade, thereby achieving more predictable pig growth and financial outcomes in the barn. These were achieved through public and private sector offerings: (a) detailed ingredient description for metabolically available amino acids (standardized ileal digestibility, **SID**) and net energy (**NE**) to facilitate valuation by ingredient and source plant, and (b) publication of the updated NRC Nutrient Requirements of Swine (2012), both of which enhanced the precision of amino acid and energy nutrition.

### Precision in ingredient nutrient description

Near Infrared Spectroscopy (**NIRS**) has proven invaluable in defining the total content of nutrient fractions so that diets could be more precisely composed. Ideally, an ingredient could be allocated to storage in a mill, according to real-time nutrient value. Alternatively, the ingredient source that provides the greatest value would be selected for priority purchase. An important advancement occurred in amino acid nutrition en route to the detail that we have today; amino acids in an ingredient could be determined by direct reading of spectral information (Fontaine *et al*., 2002). Evonik Nutrition (Essen, Germany) has proven expert, in this regard, having moved from regression equations that indirectly estimate amino acids from protein content.

During the past decade, precision nutrition was advanced even further, and in a major way, when Cargill Animal Nutrition (Minneapolis, MN, USA) allowed market access to its ‘Nutrient Reveal’ program as a product. This proprietary technology describes nutrient availability for an ingredient in the greatest detail ever afforded nutritionists in North America. Reports include (a) SID amino acids, and (b) NE; the algorithm to compute the latter is more detailed (carbohydrate fraction includes simple sugars) than the NRC (2012) was able to provide. This privately held program is the product of public and private research. Estimation of SID amino acid content is more involved than NIRS analysis, involving NIRS spectral readings related, in a proprietary manner, to an *in vitro* assay that has been aligned with the outcome of pig-based ileal digestion assay (Pilcher C, personal communication). Finally, comparative ingredient value is determined through formula runs.

### Publication of nutrient requirements for swine, 2012

Publication of the US Nutrient Requirements of Swine (NRC, 2012) advanced our precision in matching metabolically available amino acids and energy to tissue needs. The North American pork industry underwent considerable changes since the previous 10th edition (NRC 1998), and research in that period contributed a robust amount of new information for many nutrients. This publication was the product of collaboration between North American scientists, with leadership provided by the late Dr Kees de Lange being pivotal to the committee’s success. A version of his growth model was adapted for use in estimating SID amino acid and NE requirements for high lean growth pigs. Subsequent empirical data found general agreement with this model (Elsbernd *et al*., 2017). Another element of their success was that three key advances were available to the committee: (a) a SID lysine curve had been reliably established for high lean growth pigs, through extensive collaboration; (b) extensive ingredient SID amino acid data base existed and the (c) Baker (1997) ideal amino acid pattern (**IAAP**) had been extensively calibrated for five of the most limiting amino acids. The committee used several tactics to create reliability. For example, model prediction of essential amino acid needs was calibrated using a significant body of empirical research to simulate against. During the process of simulation runs to align theoretical estimates with empirical outcomes, efficiencies of absorbed amino acid (SID) for maintenance and growth were adjusted. The rigorous comparison of predicted to actual estimates included competently conducted studies with a meaningful number of pigs, reared in state-of-the-art commercial research facilities. Fortunately, the committee convened at a point in time when the major terminal lines had largely converged to similarity in carcass lean (protein to lipid content). As a result, the lysine curves (SID lysine: Mcal NE) became almost indistinguishable among lean growth genotypes.

### Ingredient net energy precision

Equations that were used to predict ingredient NE were an adaptation of those arising from INRA research (Noblet *et al*., 1994). Resulting estimates were deemed credible for core ingredients, notwithstanding the advantage of more detailed prediction equations (e.g. fractions such as sugars). Although NE is the best descriptor of absorbed energy use, concern about estimate credibility and relative diet simplicity, prior to 2008, precluded the use of the NE system by many North American nutritionists. Privately held empirical validation of ingredient NE estimates is generally not available to the public sector; however, in certain instances, estimates were provided to the committee (Dr de Lange, personal communication). This was the case for animal fat where committee impasse existed as to the accuracy of potential estimates. For the latter, growth assays were conducted, shared with the committee and later reported (Boyd *et al*., 2014 and 2015). Given the economic value of ingredient energy, we cannot overstate the importance of growth assay to validate dietary NE estimates (Schinckel *et al*., 2012), as has been routine for validating ideal amino acid patterns.

Notwithstanding the NRC NE equation being less descriptive, it was pivotal in completing the shift of North American nutritionists to NE-based formulation. The most limiting element in moving toward a more descriptive algorithm to derive ingredient NE estimates is the analytical detail required. The limitation to the public advancements is the unavoidable complexity with which different energy sources are used for maintenance as well as for lean and lipid gain. This approach considers the differing efficiencies of utilization according to both energy source (fat, protein, starch and fiber) and metabolic outcome of energy in the diet (Birkett and de Lange, 2001a, 2001b and 2001c). This is the basis for the Cargill ingredient energy prediction model.

### Empirical derivation of ideal amino acid pattern for growth

The laboratory of David Baker (1997) advanced the application of ideal amino acid pattern (**IAAP**) in North American nutrition practice by deriving the pattern through empirical assay. It proved the error of using the whole-body pattern for lysine, methionine and tryptophan. In addition, the pattern was estimated for three growth phases, thereby accounting for the changing contribution of tissue growth and maintenance to the final pattern (Fuller *et al*., 1989). Ultimately, the Baker pattern was a guiding force in the design of amino acid studies, during the lean growth transformation era (1990 through about 2012), and for amino acid research in general. A large number of empirical studies not only refined the proposed pattern for the most limiting amino acids, but they provided estimates for the NRC to simulate against. A comparison of the Baker IAAP with the resulting NRC growth model pattern is shown for two body weights (Table 1). Published data and the NRC model are in relative agreement on IAAP estimates for threonine, total sulfur amino acids and valine; some questions remain about the tryptophan ratio as most nutritionists consider the NRC estimate to be slightly low. Continued pressure to reduce diet cost and protein content of weaned pig diets, beyond the most limiting amino acids (lysine, threonine, methionine, tryptophan, valine), have since led to studies on the IAAP for presumed next limiting amino acids: isoleucine (Clark *et al*., 2017) and histidine (Cemin *et al*., 2018). The National Research Council (2012) also introduced an important concept relative to the dynamics of threonine ratio; it confirmed that the IAAP of threonine increases with BW but that it also increases with dietary fermentable fiber level.

**Table 1:** Comparison of the ideal pattern for essential amino acids (standardized ileal digestibility, **SID**) in growing pigs, expressed as a ratio to lysine level (let lysine = 1.000), for two points of growth: Baker pattern (1997) v. NRC (2012) model

	20 kg pig		80 kg pig	
	Baker^1^	NRC^2^^,^^3^	Baker^1^	NRC^2^^,^^4^
Amino acid				
Lysine	1.000	1.000	1.000	1.000
Threonine	0.650	0.572	0.700	0.608
Methionine + cysteine	0.600	0.561	0.640	0.573
Tryptophan	0.170	0.170	0.190	0.175
Valine	0.680	0.647	0.680	0.659
Isoleucine	0.600	0.521	0.600	0.529
Leucine	1.000	1.006	1.000	1.012
Histidine	0.320	0.344	0.320	0.344
Arginine	0.420	0.457	0.180	0.458
Phenylalanine + tyrosine	0.950	0.936	0.950	0.948

NE = net energy.

1 Baker patterns published in Biokyowa technical review no. 9 (1997). The ratio of each amino acid to lysine represents the minimum dietary provision required, beyond the amount that is synthesized. The relative decline in the arginine to lysine ratio for the 80 kg pig is based on arginine synthesis completely meeting the amount required for protein synthesis. The arginine ratio for 20 and 80 kg phases was considered generous because arginine is involved in immunocompetency through nitrous oxide generation.

2 US Nutrient Requirements of Swine (NRC, 2012).

3 NRC inputs: NE, 2.395 Mcal NE/kg; 4.5% fermentable fiber, gender balance, thermoneutral temperature, space not factored, model intake curve. Model protein deposition maximum estimate, 126 g/day.

4 NRC inputs: NE, 2.501 Mcal NE/kg; 6.5% fermentable fiber, gender, temperature, space as above. Model protein deposition maximum estimate, 144 g/day.

### Synthetic amino acid use

Synthetic amino acid use for pigs in North America has grown almost exponentially during the past 20 years. During this period, threonine, tryptophan and valine have been commercialized, at a cost-competitive price; isoleucine is likewise emerging for use. Profit maximization was the primary driver behind this development, with protein sources being displaced. It is not uncommon to use up to 0.65% l-lysine hydrochloride in weaned pig diets with no performance effect, provided that threonine, methionine, tryptophan and valine are also added. The North American profit model progressively expanded amino acid use, but the adoption of the NE system expanded their use further. The soybean NE : corn NE ratio does not exceed 0.82 (NRC, 2012), so the combination of corn and amino acids is a means to increasing diet energy.

North America and the European Union (**EU**) use amino acids extensively, but for different reasons. Since the primary North American driver is profit based, we have led the world in calibrating the IAAP. The European Union leads the world in amino acid use (MT of all amino acids/year) for food animal diets, but the primary driver for their growth has been legislation, with diet cost being important, but secondary. Political pressure to reduce nitrogen output led to an early demand for four to five amino acids even though diet cost initially increased. Whereas North America devoted considerable resources to IAAP validation, EU scientists devoted enormous effort to ingredient SID estimates with the results from both regions being important and complimentary.

Global growth of synthetic amino acid use has been remarkable, with the EU leading for all amino acids except for lysine, where China leads all regions. Lysine use for food animals in North America is expected to increase from 300 000 MT annually to 500 000 MT from 2010 to 2020 (Grand View Research, San Francisco, CA, USA). Although the quantity is less for North America, the rate of increase is similar to that for the EU, over the same period (500 000 to 775 000 MT/year).

## Health-promoting and -compromising properties of ingredients

The library of health-promoting ingredients expanded during the past decade. The level of peroxidized lipid products that compromise viability in weaned pigs was also shown for the first time. Some ingredients can be either health promoting or compromising; effects to be shown involve gain, efficiency of feed use, viability and medical need.

### Soybean ameliorates respiratory immune stress

A precautionary note is raised regarding the significant use of synthetic amino acids, when respiratory infection occurs. We learned that respiratory inflammatory episodes (e.g. influenza, mycoplasma pneumonia) severely impair growth and feed conversion during an infection period, and that soybean meal ameliorates these growth-suppressing effects (Johnston *et al*., 2010). This was later confirmed in a study with influenza-virus-infected pigs (Gene Gourley and Dean Boyd, unpublished research) and with weaned pigs that were deliberately infected with PRRSv (Rochell *et al*., 2015; Smith *et al*., 2019).

Soybeans contain phytochemicals that have proven to be health-promoting, including: (a) isoflavones (anti-inflammatory; Zaheer and Akhtar, 2017), (b) phenols (antioxidants; Shahidi and Ambigaipalan, 2015) and (c) saponins (suppress inflammatory mediators of tumor growth; Lima *et al*., 2017). On the basis of isoflavone and perhaps phenol contents, soybean displacement is a concern when respiratory health is compromised. It is unclear about growth-promoting aspects of soybeans (rate, composition) under conditions of low immune stress.

### Dietary xylanase improves viability

There is growing appreciation that ingredients not only supply essential energy and nutrients, but they also possess functional properties that influence animal health. This has been conclusively shown with soybeans, but the dietary enzyme, xylanase, has recently been shown to improve viability of growing pigs. Gut health improvement has held such promise, and the enzyme xylanase may be a substantive example of a measurable production outcome. While attempting to measure energy release, Zier-Rush *et al*. (2016) discovered that the addition of the carbohydrase xylanase to the diet of wean-to-finish pigs (12 to 140 kg) improved viability (3.99% to 2.39%). Although pigs were healthy, mortality declined in a dose dependent manner and by 40%. Numerous field studies have confirmed this report.

The mode of action has not yet been elucidated, but improvements in gut barrier function and improved gut microbiome profile are potential candidates. Research in poultry, having received dietary xylanase, showed promising developments for selected bacterial groups (beneficial, harmful) in the intestinal tract of broiler chicks (Vahjen *et al*., 1998). However, the results were not completely consistent; xylanase reduced enterobacteria in both luminal and GI tissue samples, and *Lactobacillus* species were increased in tissue but not luminal samples. Xylanase also appeared to enhance production of butyric acid, well known as a preferred fuel of colonic epithelial cells. *In vitro* studies have confirmed these findings and extended our understanding of a possible role of xylanase in improving gut health (Ravn *et al*., 2017).

In newly weaned pigs challenged with F18 Enterotoxigenic *E. coli* (**ETEC**; Li *et al*., 2019), xylanase was added to a diet containing a highly fermentable fiber. They observed improved gastrointestinal barrier function and reduced inflammatory intermediates, which were all associated with faster rate of gain. An earlier study by Li *et al*. (2018) evaluated a carbohydrase blend, consisting of cellulase, xylanase and β-glucanase, in pigs that were not ETEC-challenged. They reported improved growth associated with improved gut barrier integrity and reduced immune system activation. In this study, a treatment of xylanase alone did not produce the same response as the enzyme blend. Collectively, these data suggest that carbohydrases may enhance animal health and viability mediated by reductions in the permeability of the intestinal barrier, leading to reduced exposure by the pig to immunologically active molecules and toxins.

### Diet oxidative stress impairs weaned pig health and viability

Oxidative stress refers to the disproportionate production of free radicals resulting, for example, from excessive lipid peroxidation. Under conditions of normal metabolism, generation of free radicals is balanced by ubiquitous antioxidant defense mechanisms, notably those associated with ascorbic acid, glutathione, protein thiols and various scavenging enzymes (Chakraborthy *et al*., 1994). Feeding peroxidized fats to weaned pigs impaired function and morphology of the intestinal tract and growth and nutrient digestibility, but in a dose-dependent manner (Rosero *et al*., 2015b). The finding of a dose-related impairment of gut barrier function led to a field study with 2200 pigs, proving that there was a dose-related impairment of dietary lipid peroxidation on viability, medical treatment and number of excessively small pigs at the end of the nursery phase. Total antioxidant capacity and serum vitamin E decreased linearly with increasing peroxidation, which underscores the importance of lipid quality control to increasing oxidative stress in weaned pigs (Chang *et al*., 2019).

This illustrates the importance of quality control procedures (initial peroxide value, hexanal, 2, 4-decadienal). We expect that the younger pig is more susceptible to oxidative stress-induced impairment of gut barrier function than older pigs, although growth and efficiency of gain for all ages of growing pigs are impaired (Overholt *et al*., 2018). Older growing pigs appear more resilient to oxidative stress, since peroxide stress had no impact on gut integrity.

## Technologies converge to improve pathogen detection and containment

Disease pathogens are estimated to be responsible for over 20% mortality from birth to harvest in farm animals, including pigs (National Academies of Sciences, Engineering, and Medicine, 2019), which represents a substantial economic loss. If a disease cannot be prevented by vaccine or biosecurity methods, then the next tactic is detection at first infection, that is, in the incubation stage, before the pathogen spreads throughout the population. Surveillance is fundamental to control and eradicate infectious agents; however, an adequate method for early detection in populations was not available until recently. Serum sampling is a good means for detection, but it is not easy to apply, and it represents a small proportion of the herd. Testing a few pigs makes it very difficult to detect disease that resides in 1% to 2% of the population. Confirming disease more rapidly and coordinating responses within regions are major advances in North American disease response and eradication technology.

### Polymerase chain reaction methodology for pathogen detection

A milestone was achieved for swine veterinarians in North America when PCR testing of oral fluid samples converged to enable early and rapid detection of disease. Early detection of a pathogen makes it more likely to prevent pathogen movement to other sites, and beyond a geographic area. Oral fluid sampling was found to be a reliable means of PRRSv detection (Prickett *et al*., 2008). It was determined to be more accurate than serum-derived samples, because more pigs were represented. The combination of oral fluid sampling with PCR analysis was ‘game’ changing for practicing veterinarians, for three reasons (Donovan T, personal communication): (a) population surveillance (*v*. sentinels), (b) pathogen detection at an affordable price and (c) disease containment. The polymerase chain reaction technology has been refined over the past 20 years to become a rapid and affordable means of replicating a DNA sequence for subsequent assay.

### Population sampling using oral fluids collection

Oral fluids contain salivary gland liquids, but also virus particles (nucleic acids), antibodies from oral and tonsillar tissues and from blood capillaries. Introduction of this sampling method to the North American pig veterinary sector was facilitated by prior use in human medicine. Antibodies and pathogens can be detected in oral fluids collected from infected humans and animals. Oral fluid use gained prominence in the mid-1990s as a rapid and reliable means to assessing human immunodeficiency virus infection in human patients (Hodinka *et al*., 1998).

The first application of this method of population surveillance to pig veterinary practice occurred with successful surveillance of PRRSv and porcine circovirus type 2 (**PCV2**) infections in three commercial populations (Prickett *et al*., 2008). Since that time, oral fluid sampling of sow herds has been instrumental in PRRSv eradication, from specific farms, because the virus can be detected even when prevalence is below 1% to 2% of the population. Swine veterinarians in North America applied oral fluid-based testing methodologies for an increasing number of respiratory and enteric disease diagnostic applications (Bjustrom-Kraft *et al*., 2018).

Polymerase chain reaction technology is routinely used for most of the critical diseases in North America, including PRRSv, PEDv, PCV2, porcine deltacorona virus (**PDCoV**), influenza A virus. This technology is also used for other pathogens (*Mycoplasma hypopneumoniae, Actinobacillus pleuropneumoniae*, transmissible gastroenteritis virus*, Lawsonia intracellularis, Senecavirus A*). Evidence for the rapid employment of oral fluid specimens in the North American pork industry is shown by the total number of oral fluid tests performed for pathogens each year (2010 to 2018), at three major US diagnostic laboratories. Total number of pathogen tests per year increased from approximately 21 000 in 2010 to nearly 400 000 in 2018 (updated from Bjustrom-Kraft *et al*., 2018). Potential introduction of ASF to North America is an immediate concern. An oral specimen assay has been developed for detection of this pathogen (Grau *et al*., 2015), but we are not aware that the US Department of Agriculture has accepted it as an official test, to this point.

### Central reporting and communication system

Introduction of PDCoV and PEDv into North America (2013) caused veterinarians to coalesce to develop a central reporting and communication system, which will prove invaluable if other foreign diseases are introduced. This status reporting system grew rapidly after 2013, when PEDV and PDCoV viruses infected North American swine herds. This program, known as the Morrison Swine Health Monitoring Program, involves weekly reporting of PCR diagnostic results, with a weekly communiqué returned. This system allowed veterinarians to respond to the PEDV threat in a coordinated manner to limit pathogen spread. Oral fluid assay and a national scheme of communication are critical steps forward. Should ASF infect North America, the system is in place to identify and contain the threat to the farm with the end being to limit the pathogen to a locale or region.

## Supplementary material

For supplementary material accompanying this paper visit https://doi.org/10.1017/S1751731119001915. click here to view supplementary material
