# Supplementary material for: Review: innovation through research in the North American pork industry
Source: Animal. 2019 Aug 20;13(12):2951–66. doi: 10.1017/S1751731119001915 (PMC6874321; doi:10.1017/S1751731119001915)
Supplement: Supplementary file 1 [file S17517311190019155sup001.docx]

Review: Innovation through Research in the North American Pork Industry (*animal* journal)

R. D. Boyd, C. E. Zier-Rush, D.S. Rosero, J. F. Patience, K. R. Stewart, A. J. Moeser, and M. Culbertson

Supplementary Material S1. Anticipated Research Innovations in the North American Pork Industry: The Next Decade

Content Summary

This supplement is a companion to our review of innovations that have been applied in North America during the last decade (Boyd *et al*., 2019). The organizing committee of the Australian Pig Science Association requested that we also identify high impact innovations that we expect to emerge during the next decade, as a supplement the peer-reviewed paper. Anticipated innovations were distilled from discussions with seasoned experts, who interface with other scientists and production leaders in North America (Table S1). They fall into two categories: (1) high impact innovations that we expect to emerge, and (2) highly desirable solutions that are not likely to emerge, because scientific breakthrough is required to open a pathway to progress (e.g. feed efficiency, sow viability). Special attention is given to (1) precision farming, (2) feed pathogen transboundary transmission, (3) feed pathogen mitigation that is not formalin based, (4) emerging predictors of boar fertility and (5) the future of therapeutic antibiotic use for food production in North America. This communique does not address two technologies even though each would have high impact. First, the promise of gene edits to control the most serious diseases depend on public acceptance of this tactic and this may be clarified with the genomic edit for the porcine reproductive and respiratory syndrome virus (PRRSv). For that reason, we elected not to speculate on the incorporation of gene edits for other pathogens. Second, a notable omission from the list of innovations is microbiome manipulation. Notwithstanding the promise that microbiome modulation holds for health and performance improvement, knowledge of working principles is in its infancy; the gap between scientific understanding and actionable practice is daunting.

**Anticipated innovations for North American Practice in the Next Decade**

Significant innovations that we expect to emerge with high probability of being applied are summarized in Table S1. Five are briefly discussed to guide the scientific approach (smart farming technologies) to a more efficacious outcome, or to inform as to ‘state of the art’ for feed pathogen risk and mitigation; applicability of emerging predictors of boar fertility; how therapeutic antibiotic use will evolve for the North American Pork industry. A final comment involves recent breakthroughs in preserving health by mitigating ingredient challenge to enteric health (phytase superdose).

Smart farming systems to facilitate real-time process management

We expect several precision farming technologies (hereafter referred to as smart farming) to be proven effective at the farm level, during the next decade (item 1, Table S1). The North American Pork industry is embracing smart farming, with the objective being to improve productivity by using sophisticated technologies to manage execution. Interdisciplinary collaboration is the key to delivering on innovation, which means that skilled developers (public, private) must be allied with pig production operators. Seamless cooperation is an efficient means to product utility. Although there are important guiding documents for precision animal care (Banhazi *et al*., 2012; Vranken and Berckmans, 2017), we identify foundation pillars for development of employable technologies; this guidance having been derived from production operation experts in smart technology evaluation.

In North America, there is a tendency to use the terminology ‘smart farming’ as compared to ‘precision farming’, because technology can be precise, but not meet the imperative of improving labour efficiency. Four foundation pillars must be delivered on for a technology to be valuable. First, monitoring technology must integrate information to verify normality in performance (or accurate application) or abnormality. Second, there must be real time access to information, via a user-friendly interface that is intuitive and reliable. This could not be delivered unless data are captured in real time and transferred automatically over a network for instantaneous processing and concurrent interaction. Third, there must be proven reliability with financial valuation; smart technology commercial test farms are essential for proof, using the scientific method. Finally, precision processes must improve the outcome without increasing the labour requirement. Some prototype technologies require significant labour input to deliver the desired outcome (e.g. piglet crushing sensor maintenance).

Specific areas where smart farming technologies are urgently needed include animal health (detection, medical prompts), humane animal care (feed outage) and marketing precision. Technology ‘smartness’ for precise marketing of pigs is demanding, but it represents one of the most significant opportunities for revenue capture (Cheng *et al*., 2017). For this technology to be relevant (e.g., 3-D Camera sensing), it must (a) predict pig weight accurately, (b) predict number in the target weight range, and (c) it must identify the number of pigs in each pen that need to be removed. Weight prediction error of 5% (or higher) is not accurate enough for some North American systems. For example, the Hanor Company uses regression equations (includes delivered feed, bin inventory) to predict timing for first removal of pigs. Timing for successive removals is calibrated based on known carcass weight for pigs that were marketed ahead of them. Prediction error for equations is approximately 3% over the entire growth curve; this being driven to a great extent by caloric disappearance. Calibrations are made for the health status of the single sow farm from which pigs are derived (historical variation). Internal evaluation of a prototype weight prediction system resulted in meeting the 3% threshold through 90 kg, but the error rate thereafter expanded rapidly and increasingly to 14%.

*Feed pathogen risk for transboundary disease transmission*

North America is preparing for the prospect that African swine fever (ASF) may be introduced, as with porcine epidemic diarrhea virus (PEDv) in 2013. We have technology to detect ASF in a population (oral fluids assay), before clinical signs emerge (See Boyd et al., 2019). ‘Immediate’ pathogen detection, isolation and preventing transmission beyond the site are important to containment, but feed is a transmission risk for which there is no acceptable mitigation method. Although, we are unclear how PEDv was introduced to North America, developments ensued to improve our response to foreign disease. These involved gaps in our knowledge of transboundary viral transmission and acceptable means for mitigation.

The landmark study by Dee and co-workers (2018) was a key to improving response in that it addressed a significant gap in our knowledge of whether feed ingredients are a risk factor in transboundary viral transmission. This study proved that feed ingredients can be vehicles for viral transmission. They used animal and human viruses of global significance (11), and a feed pathogen transit model (China to America) to (a) identify viruses harboured by each ingredient, and (b) half-life for pathogen survival in the ingredient. Ten ingredients or feeds were tested since matrix suitability varies for each virus. Although more viruses were harboured in certain ingredients, only soybean meal harboured PRRSv, PEDv and ASF through Pacific Ocean transit. Soybean meal and these viruses are a ‘high risk combination.’ Practical conclusions are immediately applicable: (a) foreign disease status for country of origin is a deciding factor for soybean meal purchase; (b) when elimination of an ingredient or micro-ingredient is impractical (e.g. manufacture concentrated to a global region), a minimum ingredient holding time to prevent infectivity, after Ocean transit, can be computed from viral half-life estimates.

*Feed borne pathogen mitigation by organic acids*

A second important discovery was that a specific medium chain fatty acid (MCFA) composition was as effective in killing feed PEDv, as formaldehyde (Cochrane, 2015); viral load was reduced and piglet infectivity was prevented. A credible and affordable proprietary product for mitigation of feed PEDv has since emerged (Table S1, item 3). The organic acid product was scientifically developed (composition, dose) and proven to prevent infectivity in piglets (Dr. Saddoris-Clemons, personal communication, Cargill Animal Nutrition); bio-containment laboratory facilities (BSL II, BSL III) being required for in vitro and animal infectivity assay. The ability of this product to mitigate feed-borne PRRSv is unclear, however, initial results are promising in that viral load was significantly reduced. Further testing is required to determine if remaining virus is viable (potentially infective) or non-viable. Organic acids also show promise in mitigating ASF and Foot and Mouth disease. We anticipate that the ability of an organic acid or MCFA mixture to mitigate feed-borne ASF will be tested in the near-term. This will not involve an ASF surrogate virus (e.g. Senecavirus A). North America has two approved facilities for handling the virus (BSL III). The ASF virus is expected to be more difficult than PEDv because the former has a double envelope, whereas PEDv and PRRSv are single envelope viruses. Mitigation by means other than formaldehyde is required due to feed mill handling logistics and to avoid potential consumer concerns.

Improved sperm sexing and emerging predictors of boar fertility

The future of reproductive technologies will include refinement of current technologies to increase superior sire utilization. Progress will be made in low-dose sperm technology and utilization of frozen semen. Both of these technologies may require development of deep uterine insemination procedures and may lead to increased use of sex-sorted sperm, a technology that is already adopted in cattle and one that could have profound impact across multiple sectors of the swine industry (Table S1, items 4-6). The addition of single sex litters to genomic-enhanced breeding values would further elevate the rate of genetic progress.

Most of these technologies could be elevated with the ability to predict fertility in boars. Assays for sperm traits such as membrane integrity, capacitation status, mitochondrial activity, and normal chromatin structure are developed as well as functional tests such as in vitro fertilization, however, their usefulness as a predictors of fertility is limited because none of the predictive measures can be conducted at line speed of a boar stud (Flowers et al., 2013). The original intent for these assays, however, has been realized in an alternative use. They are proving useful in verifying the integrity of manufactured artificial insemination (AI) components (chemicals, disposables). The consequence of compromised AI products can be more far reaching, than subfertility of a few boars. Artificial insemination components must be proven safe to sperm based on measures of their functionality. Plastics and glues used in manufacturing of semen containers, AI catheters and other disposables used in semen collection and AI have been shown to have the potential to impact fertility (Nerin et al., 2014). Therefore, AI companies must conduct rigorous quality control testing to prevent reproductive failures from spermatoxic chemicals in manufactured components. Failure of one North American company to validate AI components led to significant infertility for respective customers in 2012; costing that customer segment millions of dollars (Boyd RD and Zier-Rush CE, unpublished observations).

*Strategic therapeutic antibiotic and complimentary enhancement*

We expect that medically important antibiotics to humans will continue to be used for therapeutic purposes in North America for animals, especially for respiratory disease (Patience, 2019) (Table S1, item 2). Wide spread antibiotic-free pig production is not possible at the present time without compromising animal wellbeing; mortality will increase. Antibiotic alternatives are more likely to be successful in controlling enteric pathogens, as compared to respiratory disease. In order for antibiotics to be effective against respiratory pathogens, they must be absorbed and travel to the lung. We are not aware of an antibiotic alternative that is absorbed from the gastrointestinal tract and lethal or inhibitory to the pathogen. The global respiratory disease library is ominous, so antibiotics are needed for a business to survive and for animal wellbeing to be preserved. The number of diseases in North America has increased and new epidemic disease is not expected to subside with the introduction of PEDv into North America (2013). However, there are non-antibiotic components that are complimentary to strategic therapeutic antibiotics and we expect this tactic to significantly displace the panacea of ‘antibiotic replacement’, in North America. When certain auxiliary components are used in combination with a therapeutic antibiotic, the outcome improves beyond what was achieved by the antibiotic alone. This was illustrated recently in weaned pigs by combining an immune enabling component (algae 1-3 β-glucan) with an antibiotic proven to be appropriate for a respiratory pathogen challenge (Boyd *et al*., 2018). The former was expected to enhance immune response while the antibiotic suppressed viral and bacterial proliferation.

Growth promoting antibiotics that are not important in human medicine (e.g. Virginiamycin), continue to be used in North America. They improve growth and (or) feed efficiency, but improving management methods reduce this benefit. Antibiotic alternatives are emerging and some tend to improve health through various mechanisms (Cheng *et al*., 2014; Boyd *et al*., 2018). We expect more pressure to document and trace outcomes to justify prescribed use, in North America; as is already occurring in Europe. Regulation of therapeutic antibiotics that are important for human medicine will continue as one would expect.

**Enteric health promotion in weaned pigs**

*Ingredient phytate destruction by dietary phytase superdose*

Dietary phytase promotes enteric health promotion of weaned pigs, because superdose levels result in almost complete destruction of ingredient phytate; a key antinutrient (Table S1, item 9). This innovation allows one to expand soybean meal use in transition diets, without impairing stool integrity under commercial conditions (triggers medication in practice) (Zier-Rush et al., 2012). This innovation is evolving to provide two other important benefits: increased dietary inositol content, which appears to be a conditionally essential nutrient for the weaned pig and improved nutrient energy and amino acid digestibility in growing pigs.

Phytase superdose refers to phytase being supplied in the diet at levels well in excess of that needed to meet the nutritional requirement for phosphorus (Holloway et al., 2019). This typically involves diet concentrations greater than 1000 FTU/kg and sometimes as high as 4000 FTU/kg. The first report of extraphosphoric effects of a phytase superdose (2000 IU/kg) in growing pigs, to our knowledge, was published by Cornell University (Pagano et al., 2007). This report incited interest in the field because bone strength and mineral content increased. Phytase superdosing of diets emerged in North America following the report of Zier-Rush and co-workers (2012) with weaned pigs; but for different reasons. Diet cost was reduced by lifting the soybean maximum; this being made possible by phytate destruction and preservation of stool integrity. A new generation of phytase enzymes subsequently emerged that were even more effective in removing phorphorus moieties from phytate and with this, a discernible improvement in gain and efficiency of gain was observed in weaned pigs (Holloway et al., 2019). Moran and co-workers (2019) probed the mechanism for this elevated performance. They observed that a phytase superdose (2500 FTU/kg) improved growth of weaned pigs for 20 d post-weaning (0.38 kg) and that diet efficiency improved for the first 10 d period (7.8%). They also observed that adding inositol to diets without phytase, improved feed efficiency equal to a phytase superdose, alone. The effect was also limited to the first 10 d. They proposed this to be the result of complete dephosphorylation of phytate (inositol phosphate position 6; IP_6_), thereby liberating myo-inositol. If this response is at least partially due to an increase in dietary inositol, then it may be a conditionally essential nutrient for weaned pigs; this apparent deficit being met through phytate destruction.

Although the growth promoting effect of a phytase superdose is smaller and less consistent with older growing pigs (Holloway *et al*., 2019), another advantage is emerging; relatively high phytase levels appear to improve nutrient digestibility. The decision by many North American nutritionists to give credit to phytase for energy and amino acid ‘uplift’ is supported by a recent publication using apparent ileal digestibility (AID) (Dersjant-Li and Dusel, 2019). They observed that diet ME increased in a linear manner with phytase addition (0, 250, 500, 1000, 2000 FTU/kg); improving 2.8%. A dose-related response was also observed for amino acids. Improved energy and amino acid digestibility coincided with improved IP_6_ digestibility; average AID amino acid improved from 76.6 to 82.9, as IP_6_ digestibility improved from 38.8 to 82.8%. Although the magnitude of energy and amino acid digestibility may differ with ingredient, treatment of ‘by-product’ ingredients with enzymes (e.g. phytase, carbohydrases), to destroy phytate and ultimately to improve starch extraction leaves little or no opportunity for nutrient ‘uplift’ when incorporated into pig diets (e.g. distiller’s dried grains with solubles). This is important to interpretation.

There is another important cautionary consideration when interpreting pig digestibility studies. The magnitude of improvement is often lower than the precision of experiments in which digestibility is being investigated, so the lack of a statistically significant improvement cannot always be interpreted as “no improvement.” The improvement in the standardized ileal digestibilty (SID) of essential amino acids, for example, may be in the range of two to three percentage points.

The chemical basis for improved digestibility in pigs and poultry, with relatively high levels of dietary phytase, is described in a review by Humer *et al*. (2015). Since phytic acid is unstable at physiological pH, it normally exists as salts with metal cations such as calcium, magnesium, potassium, manganese, iron and zinc. Amino acid digestibility is impaired because the protein – phytate complex that is refractory to pepsin digestion or by inhibition of pepsin function. Phytate directly binds protein at low pH, but also through salt linkages at a pH typical of the small intestine. Energy digestibility may be impaired by phytate binding of starch or by impairment of amylase function.

References

Banhazi T M, Lehr H, Black J L, Crabtree H, Schofield P, Tscharke M and Berckmans D 2012. Precision livestock farming: an international review of scientific and commercial aspects. International Journal of Agricultural and Biological Engineering 5, 1-9.

Boyd RD, Donovan TS, and Rush CE 2018. Strategic therapeutic antibiotic use compared to the challenge of not using antibiotics for growing pigs. Proceedings of the 18^th^ Annual Midwest Swine Nutrition Conference, 6 Sep 2018, Indianapolis, IN, USA, pp. 51-59.

Boyd RD, Zier-Rush CE, Moeser AJ, Culbertson M, Stewart KR, Rosero DS and Patience JF 2019. Review: Innovation through research in the North American pig industry. Animal, Under review.

Cheng J, Cabezon F, Que Y, Thompson NM and Schinckel AP 2017. Evaluation of the effect of the magnitude of errors in the sorting of pigs for market on the optimal market weight. The Professional Animal Scientist 33, 585-595. doi:10.15232/pas.2017-01616.

Cheng G, Hao H, Xie S, Wang X, Dai M, Huang L and Yuan Z 2014. Antibiotic alternatives: the substitution of antibiotics in animal husbandry? Frontiers in Microbiology 5, 217. doi:10.3389/fmicb.2014.00217.

Cochrane RA 2015. Chemical mitigation of microbial pathogens in animal feed and

Ingredients. MSc Thesis, Kansas State University, Manhattan, KS, USA.

Dee SA, Bauermann FV, Niederwerder MC, Singrey A, Clement T, de Lima M, Long C, Patterson G, Sheahan MA, Stoian AMM, Petrovan V, Jones CK, Jong JD, Ji J, Spronk GD, Minion L, Christopher-Hennings J, Zimmerman JJ, Rowland RRR, Nelson E, Sundberg P and Diel DG 2018. Survival of viral pathogens in animal feed ingredients under transboundary shipping models. PLoS ONE 13, e0194509. doi:10.1371/journal.pone.0194509

Dersjant-Li Y and Dusel G 2019. Increasing the dosing of a Buttiauxella phytase improves phytate degradation, mineral, energy and amino acid digestibility in weaned pigs fed a complex diet based on wheat, corn, soybean meal, barley and rapeseed meal. Journal of Animal Science (epub ahead of print, May 6 2019). doi: 10.1093/jas/skz151.

Flowers WL 2013. Triennial reproduction symposium: Sperm characteristics that limit success of fertilization. Journal of Animal Science 91, 3022-3029.

Holloway CL, Boyd RD, Koehler D, Gould SA, Li QY and Patience JF 2019. The impact of “super-dosing” phytase in pig diets on growth performance during the nursery and grow-out periods. Translational Animal Science 3, 419-428. doi:10.1093/tas/txy148.

Humer E, Schwarz C and Schedle K 2015. Phytate in pig and poultry nutrition. Animal Physiology and Animal Nutrition 99, 605-625.

Moran K, Wilcock P, Elsbernd A, Zier-Rush C, Boyd RD and van Heugten E 2019. Effects of super-dosing phytase and inositol on growth performance and blood metabolites of weaned pigs housed under commercial conditions. Journal of Animal Science (epub ahead of print, 9 May 2019). doi.org/10.1093/jas/skz156.

Nerin C, Ubeda JL, Alfaro P, Dahmani Y, Aznar M, Canellas E and Ausejo R 2014. Compounds from multilayer plastic bags cause reproductive failures in artificial insemination. Scientific Reports 4, 4913. doi.org/10.1038/srep04913.

Pagano AR, K. Yasuda, K. R. Roneker, and, T. D. Crenshaw, X. G. Lei. 2007. Supplemental Escherichia coli phytase and strontium enhance bone strength of young pigs fed a phosphorus-adequate diet. J. Nutr. 137:1795-1801.

Patience, JF 2019. Feeding and management for antibiotic reduced/free pork production. In Proceedings of the 2019 Ensminger Pig Symposium, 22-23 May 2019, Benoni, South Africa, pp. 1-10

Vranken E and Berckmans D 2017. Precision livestock farming for pigs. Animal Frontiers 7, 32-37. doi:10.2527/af.2017.0106.

Zier-Rush CE, Smith S, Palan R, Steckel J, Wilcock P and Boyd RD 2012. Effect of a supra-phytase level on post-weaning growth and stool firmness of pigs fed low or high soybean meal diets. Proceedings of the 12th International Symposium on Digestive Physiology of Pigs, 21 May-1 Jun 2012, Keystone, CO, USA 1086, pp. 72.

**Table S1.** Summary of anticipated innovations expected to be applicable to North American Pork production in the next 10-12 years. Research is anticipated to derive from global innovation. Item 2 is North America policy based on the direction of practice and expected public policy.

| **Item** | **Main effects on production solution** |
| --- | --- |
| 1. Smart farm technology^1^ | Sensory devices: Pre-wean piglet protection from sow; RFID sow medical records for outcomes; |
|  | precision sow pregnancy feeding guided by camera body caliper; feed bin inventory (< 6% error), |
|  | feed bridging alerts; camera (3-D) weight for performance and market weight prediction; |
| 2. Therapeutic antibiotic use^1^ | Medically important antibiotics continued but outcomes documented to evaluate effectiveness; |
|  | Auxiliary components identified as enhancing outcome to antibiotics alone. |
| 3. Feed pathogen mitigation^2^ | Specific MCFA, combination and form (free acid, monoglyceride) proven effective against PRRSv, |
|  | PEDv, ASF in biosafety facilities through animal infectivity assay. |
| 4. Deep uterine insemination^1^ | Methods proven for genetic nucleus use with little or no loss in farrow rate, litter size |
| 5. Emerging predictors of boar fertility^1^ | 1-2 components may become operable at boar stud line speed, but several are now appropriate |
|  | for artificial insemination supply companies to validate that supplies do not compromise sperm. |
| 6. Sperm sexing^1^ | Rate and accuracy improved for genetic nucleus use and perhaps production nucleus sites. |
| 7. NIRS prediction for amino acid availability^2^ | Public access to real-time prediction of soybean meal amino acid standardized ileal digestibility via |
|  | AusScan technology developed by the Australian Pork CRC research program. |
| 8. NIRS technology for abattoir carcass sortation^2^ | Loin intramuscular fat based sortation from population for selected customer segments |
| 9. Enteric health promotion^1,2^ | Medium and long chain fatty acid components expected to reduce gastrointestinal challenge to |
|  | bacteria and viruses; by envelope disruption or pH reduction. Phytase superdosing to destroy Ingredient phytate alleviates enteric challenge in weaned pigs; promote nutrient digestibility. |
| **Limited to Incremental improvement unless Understanding of Biology Improves** | |
| 10. Sow robustness to disease | Subclinical disease expected to be a significant cause (e.g. circovirus). |
| 11. Genetic means to improved feed efficiency |  |
| 12. Vaccine effectiveness for catastrophic disease^3^ | Advances in animal vaccinology have followed advances in human vaccinology. |
| 13. Microbiome manipulation | Improve gain, feed efficiency, and viability are possible outcomes of appropriate manipulation. |

^1^ Discussed in text. RFID, radio-frequency identification.

^2^ NIRS, near-infrared spectroscopy; PRRSv, porcine reproductive and respiratory syndrome virus; PEDv, porcine epidemic diarrhea virus; ASF, African Swine Fever, MCFA, medium chain fatty acids, developed to be lethal or severely suppressive to proliferation and at cost that is not prohibitive to use. BSL III represents biosafety level III laboratories.

^3^ Animal vaccines are normally the beneficiary of advances in human vaccinology. No indication of sufficient advances in areas such as core antigen subunit technology which might improve control of diseases such PRRSv, Influenza and ASF.
